# ChatGPT versus DeepSeek in head and neck cancer staging and treatment planning: guideline-based study

**DOI:** 10.1007/s00405-025-09524-4

**Published:** 2025-06-17

**Authors:** Burcu Vural Camalan, Sumeyra Doluoglu, Nazlim Hilal Taraf, Mehmet Murat Gunay, Samet Ozlugedik

**Affiliations:** Department of Otorhinolaryngology Head and Neck Surgery, University of Health Sciences Ankara Etlik City Hospital, Varlık Mahallesi, Halil Sezai Erkut Caddesi, No:5, 06170 Yenimahalle, Ankara Turkey

**Keywords:** Artificial intelligence, ChatGPT, DeepSeek, Head and neck cancer, Large language models

## Abstract

**Purpose:**

This prospective simulation study was conducted to evaluate and compare the performance of ChatGPT (o1, 2023) and DeepSeek (V3, 2024) in staging and treatment planning for head and neck cancers.

**Methods:**

This prospective simulation study was conducted in March 2025 to evaluate and compare the performance of two advanced artificial intelligence (AI) models, ChatGPT (o1, 2023) and DeepSeek (V3, 2024), in clinical decision-making for head and neck malignancies. A total of 50 hypothetical, guideline-based clinical scenarios were carefully designed in English by two otorhinolaryngologists in alignment with the National Comprehensive Cancer Network® (NCCN®) Guidelines Version 2.2025.

**Results:**

In the overall analysis of treatment planning performance, DeepSeek (V3, 2024) demonstrated statistically superior accuracy compared to ChatGPT (o1, 2023) (*p* = 0.04). Both models showed comparable performance in tumor staging (*p* = 0.83). Both DeepSeek (*p* = 0.0001) and ChatGPT (*p* = 0.02) were statistically successful in respect of staging accuracy and providing fully correct answers on the subject of treatment.

**Conclusion:**

Although DeepSeek V3 demonstrated promising capability for clinical decision support in head and neck oncology, these artificial intelligence tools cannot replace multidisciplinary tumor boards. However, they can significantly streamline clinical workflows by rapidly organizing patient data, thereby enhancing board efficiency. Future efforts should prioritize the development and integration of secure, institution-specific, local large language models tailored for oncological decision-making.

**Supplementary Information:**

The online version contains supplementary material available at 10.1007/s00405-025-09524-4.

## Introduction

Head and neck cancers (HNC) encompass a diverse array of malignancies characterized by their complex anatomy and variable biological behavior. Accurate staging and effective treatment planning are crucial, as they directly impact patient prognosis, functional outcomes, and quality of life [1]. Therapeutic strategies are often developed within multidisciplinary tumor boards (MDTs), where decisions are guided by established clinical frameworks, such as the National Comprehensive Cancer Network (NCCN) Guidelines [2, 3].

With recent advances in deep learning (DL) and natural language processing (NLP) in particular, there has been a significant increase in the application of artificial intelligence (AI) in clinical fields. Of these AI resources, tools such as ChatGPT and DeepSeek are frequently used as they have demonstrated the ability to rapidly process and synthesize complex clinical data [4, 5]. The incorporation of these tools in MDTs may help reduce logistical barriers, shorten discussion times, and facilitate more focused, patient-centered decision-making [6–8].

ChatGPT, designed for general-purpose dialogue generation, has shown promise in medical applications, particularly in diagnostic tasks. However, concerns remain regarding its reliability in recommending treatments that align with clinical guidelines and its propensity to generate inaccurate or unverifiable citations [9, 10]. A recent systematic review further highlighted ChatGPT limitations in clinical reasoning beyond diagnostic suggestions, noting a decline in accuracy when proposing appropriate investigations or management plans, as well as the frequent occurrence of hallucinated references [11]. In contrast, DeepSeek employs a Mixture-of-Experts (MoE) architecture, selectively activating task-specific modules to enhance reasoning efficiency, especially in complex, logic-based tasks [9]. Although numerous studies have evaluated individual large language models (LLMs), there are few direct comparisons of different models in the field of head and neck oncology. While ChatGPT 4.0 has shown a reasonable alignment with MDT decisions, there are still gaps in oncological nuance and patient-specific recommendations [12, 13].

This prospective simulation study was conducted to evaluate and compare the performance of ChatGPT (o1, 2023) and DeepSeek (V3, 2024) in staging and treatment planning for head and neck cancers. To the best of our knowledge, while the diagnostic capabilities of ChatGPT in head and neck cancers have been previously examined, this is the first study to directly compare it with DeepSeek V3 in this context.

## Materials and methods

This prospective simulation study was conducted in March 2025 to evaluate and compare the performance of two advanced artificial intelligence (AI) models, ChatGPT (o1, 2023) and DeepSeek (V3, 2024), in clinical decision-making for head and neck malignancies. A total of 50 hypothetical, guideline-based clinical scenarios were carefully designed in English by two otorhinolaryngologists in alignment with the National Comprehensive Cancer Network® (NCCN®) Guidelines Version 2.2025 [2, 3]. These scenarios encompassed eleven distinct head and neck cancer subsites and included sufficient clinical and radiological detail to allow for accurate staging and treatment planning. Institutional review board approval was not required, as the study did not involve human subjects or any identifiable patient data.

To ensure standardization across the models, both systems were initially presented with the following instruction:

“I have prepared several head and neck cancer cases for evaluation. First, I will present each case, and I would like you to determine the tumor stage based on the provided clinical and imaging findings. After staging, I will request an appropriate treatment plan based on current guidelines and best practices. Do you understand the approach?”

Each case was then presented to both AI models. Initially, they were asked to determine the tumour stage; this was followed by a request for a guideline-concordant treatment recommendation. All the responses were independently recorded by two otorhinolaryngologists and evaluated against the NCCN Guidelines Version 2.2025 [2, 3]. According to the TNM staging defined by the American Joint Committee on Cancer (AJCC), the tumour staging responses were categorized as either “correct” or “incorrect”. Treatment recommendations were rated based on the NCCN guidelines according to the clinical experience of two specialist otorhinolaryngologists, using a three-tiered scale: “incorrect”, “partially correct” or “fully correct”.

### Statistical analyses

Descriptive statistics were used to summarize the results and were presented as absolute numbers and percentages. The accuracy of responses from ChatGPT (o1, 2023) and DeepSeek (V3, 2024) was compared using chi-square tests for categorical variables. A *p*-value < 0.05 was considered statistically significant. All analyses were conducted using PASW Statistics version 18 software (SPSS Inc., Chicago, IL, USA).

## Results

**Fig. 1:**
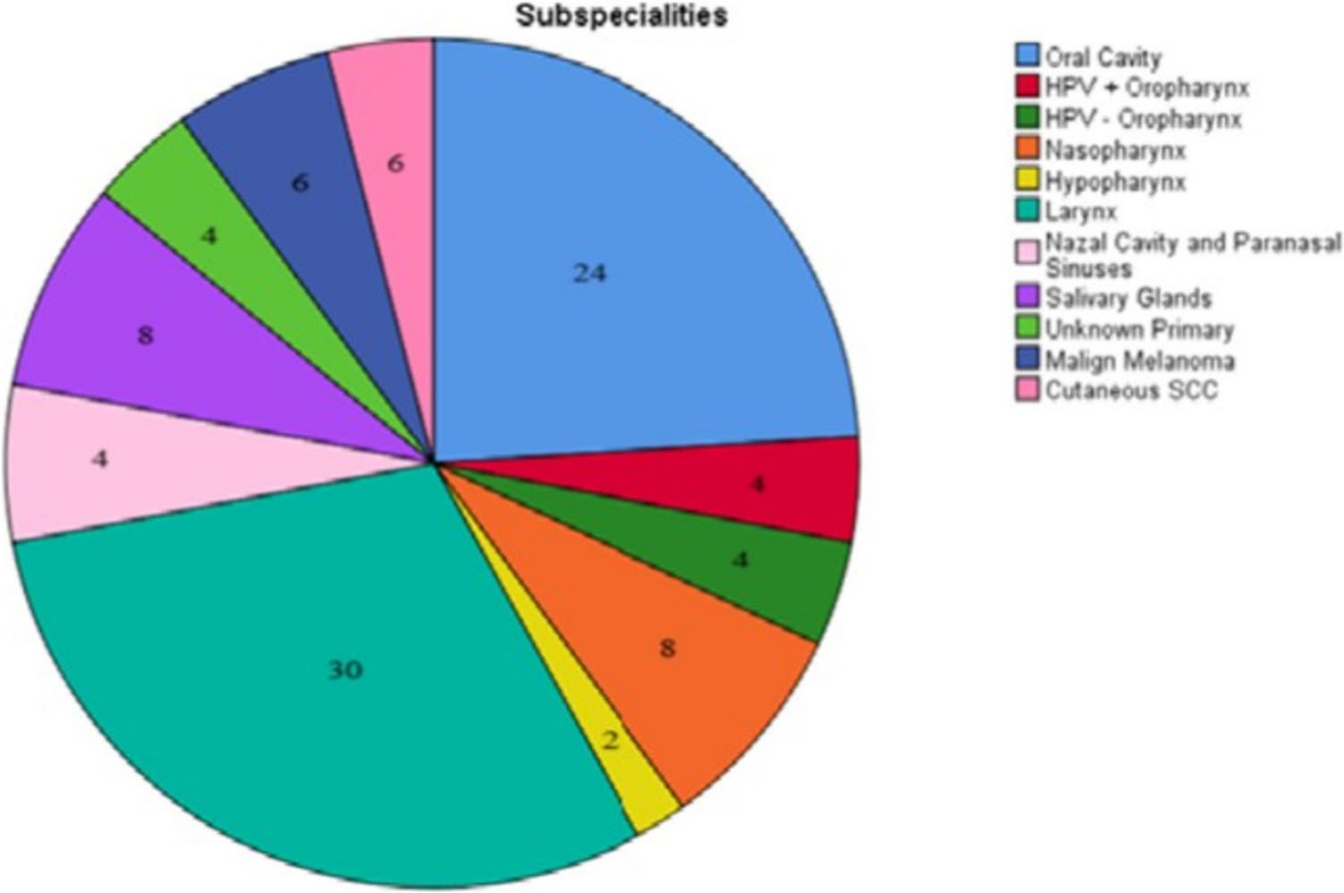
Distribution of clinical scenarios according to the head and neck cancer subsites

A total of 50 hypothetical clinical scenarios covering 11 distinct subsites of head and neck cancers were evaluated in this study. The summary of clinical cases were shown in Table 1. Of these, 15 (30%) cases involved laryngeal cancers and 12 (24%) involved oral cavity cancers. The distribution of cancer subsites illustrated in Fig. 1. An example of the clinical scenarios together with the corresponding staging and treatment responses from both AI models are summarized in Table 2.

**Table 1:** The summary of clinical case scenarios

ID	Age	Gender	cTx	cNx	cMx	Subsite of the tumour	Recommendation of the NCCN
1	63	M	3	0	0	Larynx (Transglottic)	Total Laryngectomy + Bilateral Level 2–3–4 Neck Dissection
2	66	F	is	0	0	Larynx (Glottic)	Definitive Radiotherapy (RT)
3	63	F	2	0	0	Oral Cavity (Hard palate)	Inferior Maxillectomy
4	75	F	2	0	0	Salivary Gland (Parotid)	Total Parotidectomy + Level 1–2–3 Neck Dissection
5	50	M	2	0	0	Larynx (Glottic)	Definitive Radiotherapy (RT)
6	49	M	1	0	0	Oral Cavity (Lower lip, right)	Tumour Resection + Right Level 1–2 Neck Dissection
7	35	M	3	2	1	Nasopharynx	Chemoradiotherapy (CRT) following induction chemotherapy (ICT)
8	65	M	4a	0	0	Larynx (Transglottic)	Total Laryngectomy + Bilateral Level 2–3–4 Neck Dissection + Subtotal Thyroidectomy + Bilateral Level 6 Neck Dissection
9	67	M	3	1	1	Oropharynx HPV (+)	Chemoradiotherapy (CRT)
10	31	M	2	2	0	Nasopharynx	Chemoradiotherapy (CRT) following induction chemotherapy (ICT)
11	60	M	2	0	0	Oral Cavity (Lower lip, left)	Tumour Resection + Left Level 1–2–3 Neck Dissection
12	64	M	1	0	0	Oral Cavity (Tongue, right)	Tumour Resection + Level 1 A Right Level 1B 2 A,2B,3 Neck Dissection
13	50	F	4	0	0	Nazal Cavity and Paranasal Sinuses (Clivus Chordoma)	Surgery + Adjuvan Radiotherapy (RT)
14	58	M	3	2a	0	Hypopharynx (left)	Gastric Pullup Procedure + Left Modified Radical Neck Dissection + Right Level 1–2–3 Neck Dissection OR Chemoradiotherapy (CRT)
15	87	F	3	0	0	Cutaneous (left preauricular region)	Tumour Resection + Parotidectomy + Level 1–2 Neck Dissection
16	73	M	1	0	0	Larynx (Glottic)	Transoral Surgery
17	68	F	2	0	0	Oral Cavity (Lower lip, left)	Resection and Reconstruction with a local flap
18	78	M	1	0	0	Cutaneous (left auricula)	Tumour Resection
19	71	M	1	2c	0	Larynx (Supraglottic)	Chemoradiotherapy (CRT)
20	67	M	3	1	0	Oropharynx HPV (-)	Chemoradiotherapy (CRT) following induction chemotherapy (ICT)
21	74	M	4	0	0	Larynx (Subglottic)	Total Laryngectomy + Bilateral Level 2–3–4 Neck Dissection + Bilateral Level 6 Neck Dissection + Subtotal Thyroidectomy + Left Inferior Parotidectomy
22	54	M	3	0	0	Larynx (Transglottic)	Chemoradiotherapy (CRT)
23	73	M	3	1	0	Larynx (Supraglottic)	Chemoradiotherapy (CRT)
24	74	M	2	0	0	Oral Cavity (Tongue, right)	Tumour Resection + Reconstruction
25	70	M	2	0	0	Oral Cavity (Floor of the mouth)	Floor of Mouth Resection + Bilateral Level 1–2–3–4 Neck Dissection + Reconstruction with Anterolateral Thigh Free Flap
26	74	F	4a	0	0	Oral Cavity (Hard palate, left)	Left Inferior Maxillectomy + Left Level 1–2–3 Neck Dissection
27	60	M	3	2c	0	Larynx (Supraglottic)	Chemoradiotherapy (CRT)
28	65	M	3	0	0	Larynx (Supraglottic)	Transoral Surgery or Supraglottic Laryngectomy + Bilateral Level 2–3–4 Neck Dissection
29	60	M	4	2c	0	Oropharynx HPV (-)	Chemoradiotherapy (CRT)
30	68	M	0	2c	0	Unknown primary	Chemoradiotherapy (CRT)
31	63	M	4	2	0	Oropharynx HPV (+)	Chemoradiotherapy (CRT) following induction chemotherapy (ICT)
32	50	M	4a	0	1	Oral Cavity (Buccal region, right)	Systemic Chemotherapy
33	82	F	3	0	0	Mucosal Melanoma (left nasal)	Surgery + Adjuvan Chemoradiotherapy (CRT)
34	73	M	4a	0	0	Nazal Cavity and Paranasal Sinuses (Maxillary Sinus)	Chemoradiotherapy (CRT) following induction chemotherapy (ICT)
35	38	F	2	0	0	Salivary Gland (Submandibular gland)	Submandibular Gland Excision
36	57	M	0	3b	0	Unknown primary	Chemoradiotherapy (CRT) following induction chemotherapy (ICT)
37	49	M	3	1	0	Nasopharynx	Chemoradiotherapy (CRT) following induction chemotherapy (ICT)
38	55	M	3	1	0	Oral Cavity (Tongue, right)	Surgery + Adjuvan Chemoradiotherapy (CRT)
39	55	M	2	3b	0	Nasopharynx	Chemoradiotherapy (CRT) following induction chemotherapy (ICT)
40	68	M	4b	2	0	Larynx (Transglottic)	Salvage Surgery + Adjuvan Chemoradiotherapy (CRT)
41	41	M	4b	3b	0	Larynx (Supraglottic)	Chemoradiotherapy (CRT) following induction chemotherapy (ICT)
42	82	M	2	2b	0	Salivary Gland (Parotid)	Surgery + Adjuvan Chemoradiotherapy (CRT)
43	84	M	4b	3b	0	Oral Cavity (Buccal region, right)	Unresectable Head and Neck Tumor – Re-evaluation After Induction Chemotherapy (ICT)
44	71	M	4a	2c	1	Larynx (Transglottic)	Palliative Systemic Chemotherapy
45	57	F	4	0	0	Mucosal Melanoma (left nasal)	Systemic Chemotherapy
46	50	M	3	0	0	Mucosal Melanoma (left nasal)	Surgery + Adjuvan Chemoradiotherapy (CRT)
47	47	M	2	0	0	Nazal Cavity and Paranasal Sinuses (Nasal cavity)	Tumour Resection
48	17	M	2	0	0	Oral Cavity (Hard palate)	Tumour Resection
49	14	F	1	0	x	Salivary Gland (Parotid)	Total Parotidectomy
50	60	M	4b	0	0	Larynx (Transglottic)	Palliative Systemic Chemotherapy

Abbreviations: *HPV* Human Papilloma Virus; *CRT* Chemoradiotherapy; *ICT* Induction chemotherapy; *RT* Radiotherapy

**Table 2:** Sample clinical scenario with AI-generated response and reference answer based on NCCN guidelines

Question: A 49-year-old male patient presented with a one-year history of a sore on the lower lip. Clinical examination revealed an approximately 1 × 1 cm dry vegetating lesion located on the right lateral aspect of the midline of the lower lip, with no palpable lymphadenopathy detected in the neck. Other ENT examinations were unremarkable. Histopathological analysis of the incisional biopsy confirmed keratinized squamous cell carcinoma. Imaging studies showed no pathological findings. What is the stage of tumour? What is the treatment plan of the tumour?					
Staging			Treatment		
NCCN	ChatGPT o1, 2023	DeepSeek V3, 2024	NCCN	ChatGPT o1, 2023	DeepSeek V3, 2024
T1N0M0 Lip SCC	**Tumor Staging (AJCC 8th Edition for Lip Cancer)** **Key Clinical and Imaging Findings:** **Tumor Location:** Right lateral aspect of the midline of the lower lip **Size: 1 × 1 cm** **No Deep Invasion:** No bone invasion or extension to adjacent structures **No Lymph Node Involvement:** No palpable cervical lymphadenopathy (N0) **No Distant Metastases (M0)** **Tumor Stage:T1:** Tumor ≤ **2 cm** in greatest dimension without extraparenchymal extension **N0:** No regional lymph node metastasis **M0:** No distant metastasis **Final Stage: T1N0M0 (Stage I – AJCC 8th Edition for Lip Cancer)**	**Case Staging** **Primary Site**: Lower lip (oral cavity) **Histology**: Keratinized squamous cell carcinoma **TNM Classification (AJCC 8th Edition for Oral Cavity) **: - **T (Tumor)**:—Tumor size ≤ 2 cm (1 × 1 cm) with no deep invasion (e.g., bone, muscle, or skin beyond the lip) - **T1** - **N (Nodes)**: No clinical or radiologic lymphadenopathy → **N0** - **M (Metastasis)**: No distant metastasis → **M0** **Overall Stage**: **Stage I** **(T1N0M0)**	Tumour Resection + Right Level 1–2- Neck Dissection	**Treatment Plan** For **early-stage (T1N0) squamous cell carcinoma of the lower lip**, the goal is **curative treatment with functional and aesthetic preservation** **1. Surgical Excision (Preferred)** **Wide local excision with primary closure** **Recommended margin:** At least **5 mm** for well-differentiated SCC (10 mm if poorly differentiated) **Reconstruction:** If small, direct primary closure is feasible. If larger, **local advancement flaps (e.g., Karapandzic, Abbe, or Estlander flaps)** may be needed **No elective neck dissection** (since **T1N0 SCC of the lip has a low risk of lymphatic spread**) **2. Radiotherapy (Alternative for Non-Surgical Candidates)** **Definitive External Beam Radiotherapy (EBRT) or Brachytherapy** **Indications:** Medically inoperable patients, Patients unwilling to undergo surgery **Dose: 50–66 Gy** in 25–33 fractions **Considerations:** Less preferred than surgery due to longer treatment duration and potential functional impairment **Follow-up & Surveillance Plan** **Regular follow-up every 3 months for the first 2 years, then every 6 months for 5 years** **Monitor for local recurrence and lymph node involvement (cervical ultrasound if needed)** **Patient education on sun protection and risk factor modification (e.g., tobacco cessation)** **Summary of Recommended Treatment Approach:** ✅ **Wide local excision with clear margins (preferred approach)** ✅ **No elective neck dissection (unless high-risk features are found in final pathology)** ✅ **Radiotherapy only for inoperable patients** ✅ **Long-term follow-up to monitor for recurrence** Would you like details on surgical techniques or reconstruction options for this case?	**Treatment Recommendations** **Primary Treatment**: 1. **Surgical Excision**: **Wide local excision** with ** ≥ 5 mm margins** (preferred for small, localized T1 lesions) **Intraoperative frozen section** to confirm clear margins **Reconstruction**: Primary closure or local flap (e.g., wedge resection) based on defect size 2. **Radiation Therapy (RT)**: Alternative if surgery is contraindicated or patient preference **Dose**: 60–66 Gy in conventional fractionation **Key Considerations**: - **Margins**: Clear margins are critical to minimize recurrence risk - **Cosmetic/Functional Outcomes**: Lower lip lesions often heal well with surgical resection - **No Elective Neck Treatment**: Nodal metastasis risk is < 5% in T1N0 lip cancer **Adjuvant Therapy**: - **Not indicated** if margins are clear and no high-risk features (e.g., perineural invasion, lymphovascular invasion) NCCN Guidelines Summary**: - **Stage I Lip Cancer**: - **Surgery** is first-line for resectable tumors - **RT** for non-surgical candidates Final Recommendation**: - Proceed with **wide local excision** and margin assessment. If margins are clear, no further treatment is needed - If margins are positive, re-excision or adjuvant RT is recommended Follow-Up**: - **Clinical exams** every 3–6 months for 2 years, then annually - **Sun protection counseling** (lip SCC is often UV-related) - **Tobacco cessation** if applicable Rationale Against Systemic Therapy**: No role for chemotherapy or immunotherapy in localized T1N0 disease Would you like to discuss another case?

### Overall model performance

In tumor staging, ChatGPT (o1, 2023) correctly identified the stage in 30 of 50 cases (60%), and DeepSeek (V3, 2024) was correct in 31 cases (62%). The difference was not statistically significant (*p* = 0.83). The overall staging accuracy was similar between ChatGPT (60%) and DeepSeek (62%), whereas the distribution of staging errors differed. Both models failed to correctly classify the same 10 cases, and ChatGPT and DeepSeek each made additional unique errors in 10 and 9 other scenarios, respectively. The list of overlapping and non-overlapping misclassified cases is provided in Supplementary Table 1 and Supplementary Table 2, respectively.

In respect of the treatment recommendations, ChatGPT provided 31 (62%) fully correct and 19 (38%) partially correct responses. DeepSeek produced 40 (80%) fully correct and 10 (20%) partially correct responses. Neither model generated any incorrect treatment responses. The difference in treatment accuracy between the two models was statistically significant (*p* = 0.04). The overall statistical outcomes are provided in Table 3.

**Table 3:** Comparative accuracy of ChatGPT and DeepSeek in tumour staging and treatment planning across 50 clinical scenarios

	Staging (*n* = 50)		Treatment (*n* = 50)		*p *value
	Correct	Incorrect	Fully correct	Partially correct	
ChatGpt o1	30 (60%)	20 (40%)	31 (62%)	19 (38%)	0.83*
DeepSeek V3	31(62%)	19 (38%)	40 (80%)	10 (20%)	0.04*

^*^Pearson Chi-Square

When the treatment and staging responses of ChatGPT were compared with each other in respect of accuracy, a statistically significant difference was determined (*p* = 0.02). The comparison of the accuracy of the staging and treatment responses of DeepSeek also revealed a statistically significant difference (*p* = 0.0001).

### Subgroup analyses

In oral cavity cancer scenarios (*n* = 12), ChatGPT correctly staged 7 (58.3%) cases and failed in 5 (41.7%). DeepSeek correctly staged 8 (66.7%) and failed in 4 (33.3%). When the two AI models were compared in respect of staging, there was no statistically significant difference (*p* = 0.67). In treatment planning, ChatGPT provided 6 (50%) fully correct and 6 (50%) partially correct responses. DeepSeek delivered 8 (66.7%) fully correct and 4 (33.3%) partially correct responses. When the two AI models were compared in respect of treatment, there was no statistically significant difference (*p* = 0.40).

Among the laryngeal cancer scenarios (*n* = 15), ChatGPT correctly staged 10 (66.7%) cases, and DeepSeek achieved correct staging in 8 (53.3%). When the two AI models were compared in respect of staging, there was no statistically significant difference (*p* = 0.45). Regarding treatment, ChatGPT gave 10 (66.7%) fully correct and 5 (33.3%) partially correct responses. DeepSeek provided 11 (73.3%) fully correct and 4 (26.7%) partially correct responses. When the two AI models were compared in respect of treatment, there was no statistically significant difference (*p* = 0.69).

In the staging of the 3 scenarios of sinonasal cancer, both AI models produced 2 correct and 1 incorrect response. In the selection of treatment, ChatGPT provided 2 fully correct and 1 partially correct response, and DeepSeek gave 3 fully correct responses. In the staging of the 3 scenarios prepared for mucosal melanoma, DeepSeek provided a correct response for all 3, and ChatGPT gave 2 correct and 1 incorrect response. For the questions related to treatment, DeepSeek provided a fully correct response to all 3 questions, and ChatGPT gave 1 fully correct and 2 partially correct responses. For the 2 scenarios prepared for unknown-origin primary cancers, DeepSeek provided correct staging for both, and ChapGPT gave 1 fully correct and 1 partially correct response. For the 8 questions including these rarely seen sinonasal cancers, mucosal malignant melanomas, and head and neck tumours of unknown origin, there was determined to be a statistically significant difference between the two AI models in respect of the fully correct responses for treatment (*p* = 0.02).

**Fig. 2:**
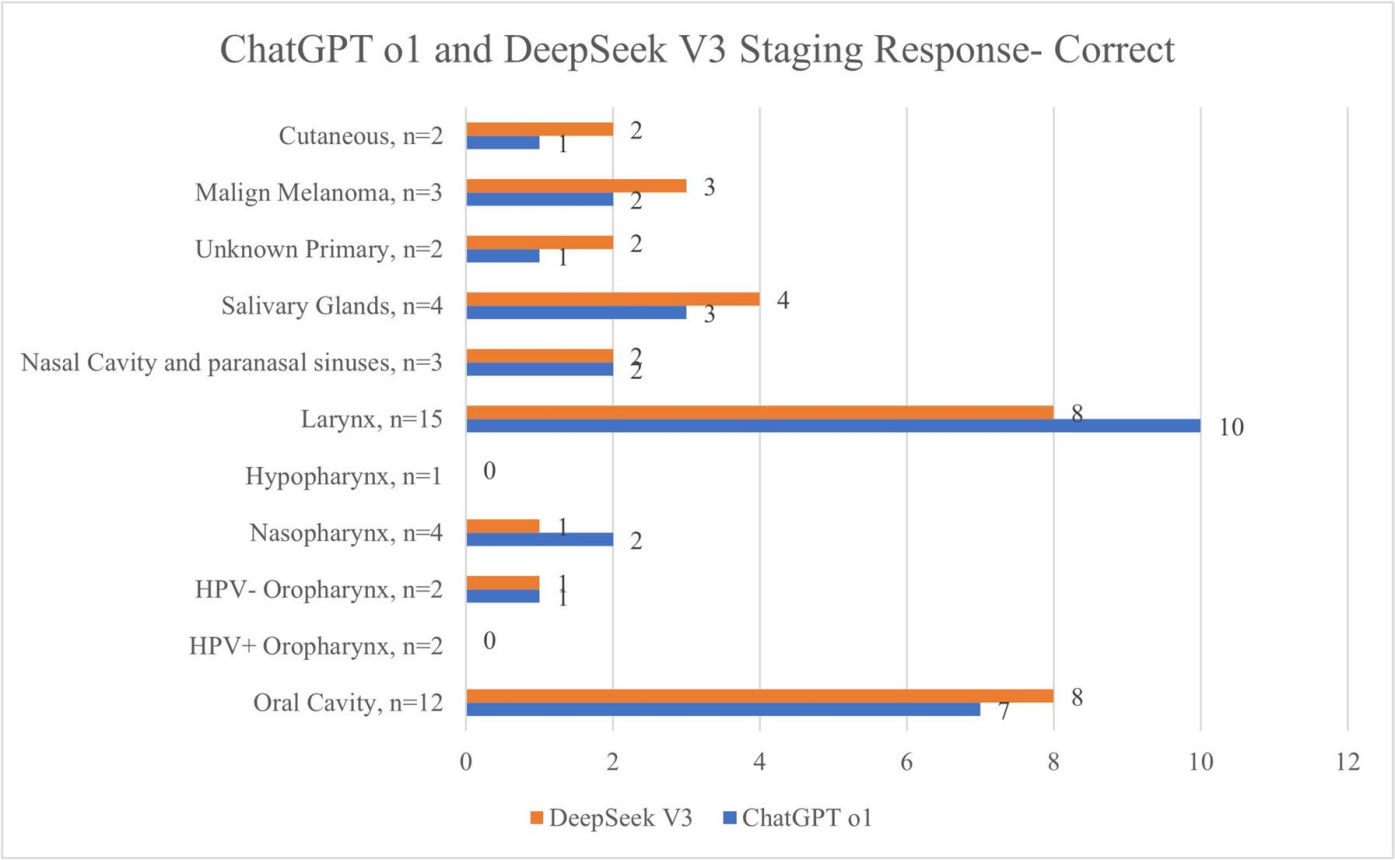
Comparisons of the staging accuracy of the models according to the head and neck cancer subsites

**Fig. 3:**
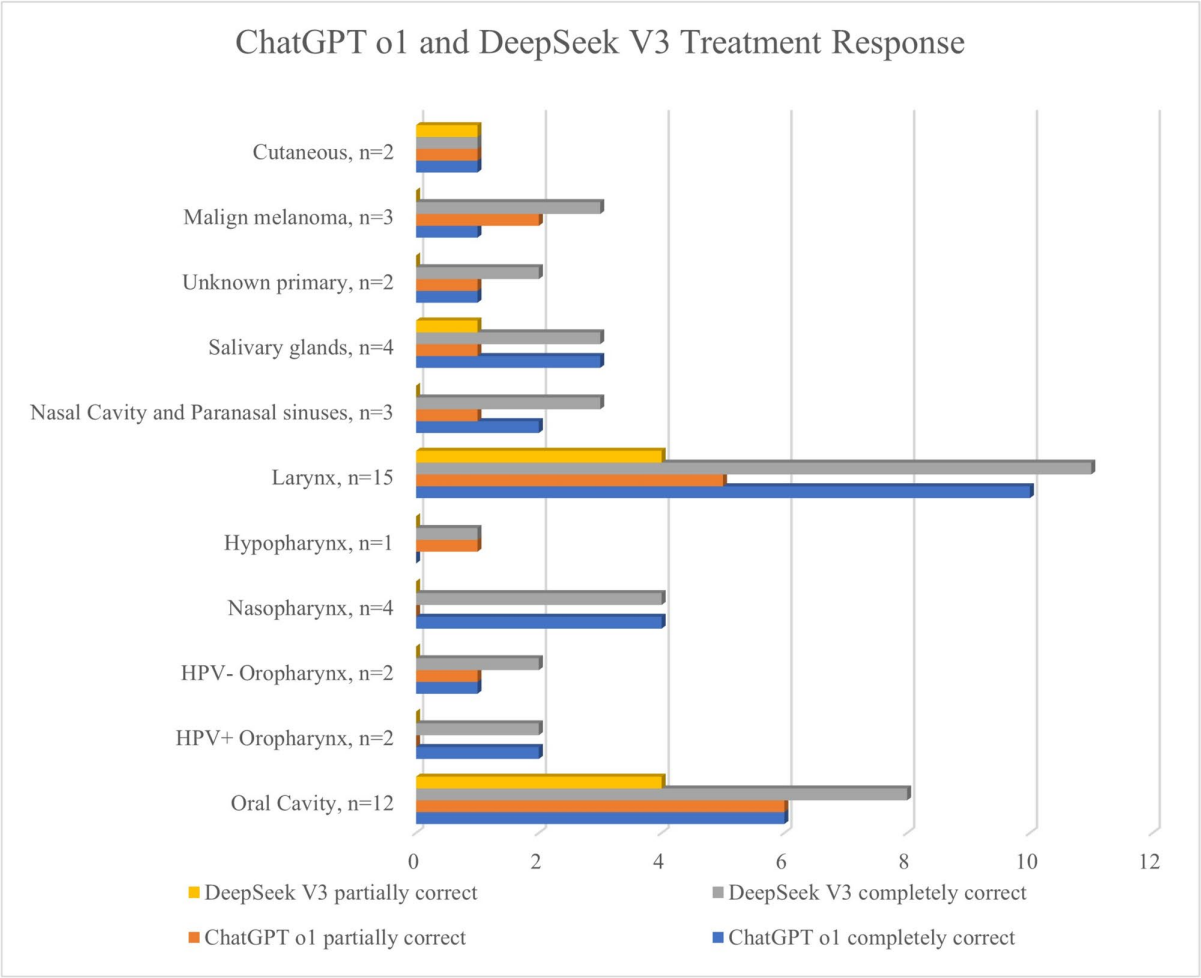
Comparisons of the treatment accuracy of the models according to the head and neck cancer subsites

When comparisons were made of the fully correct responses given by ChatGPT in respect of treatment for these rarely seen cancers (8 questions) and the other more common cancers (42 questions), no statistically significant difference was determined (*p* = 0.44). Similarly for DeepSeek, no statistically significant difference was determined in the comparisons of the fully correct responses in respect of treatment for these rarely seen cancers (8 questions) and the other more common cancers (42 questions) (*p* = 0.12). The performance trends across all the cancer subsites are visualized in Figs. 2 and 3, showing the accuracy of staging and treatment responses, respectively, stratified by subsite.

## Discussion

In recent literature, LLMs have been applied in the context of head and neck cancers and MDTs to assess their potential in supporting clinical decision-making [12, 13]. A previous study explored the use of ChatGPT and Claude 3 for primary head and neck cancer cases, reporting promising feasibility in assisting tumor board recommendations [1]. In addition, more recent research has evaluated web-based (ChatGPT-4o) and locally hosted (LLaMA 3) LLMs, reflecting a growing methodological interest in AI-supported decision frameworks and data protection concerns in oncology practice [14].

To the best of our knowledge, this study is the first to directly compare ChatGPT-01 and DeepSeek V3 in the context of tumor staging and treatment planning for head and neck cancers. By utilizing structured clinical scenarios derived from the NCCN Guidelines, this research provides a novel comparative framework for evaluating the accuracy and clinical relevance of publicly accessible LLMs in the specific setting of head and neck cancer subsites. Beyond assessing accuracy, the primary aim was to explore whether these models could reliably support MDT workflows by accelerating tumor staging and enabling more structured, concise case presentations, thereby ultimately contributing to more time-efficient and focused board discussions.

In the overall analysis of treatment planning performance, DeepSeek (V3, 2024) demonstrated statistically superior accuracy compared to ChatGPT (o1, 2023) (*p* = 0.04). While both models exhibited comparable performance in tumor staging (*p* = 0.83). DeepSeek provided fully correct treatment recommendations in 80% of cases, significantly outperforming ChatGPT, which achieved 62% (*p* = 0.04). These results align with previous findings where DeepSeek outperformed proprietary models in other high-level tasks, including mathematical reasoning and code generation [15]. However, model performance may vary across clinical domains. In a recent pediatric diagnostic study by Mondillo et al., ChatGPT o1 achieved a diagnostic accuracy of 92.8%, significantly outperforming DeepSeek-R1, which scored 87.0% (*p* = 0.0001) [16]. In contrast, the current study data suggest that the logic-intensive architecture of DeepSeek may be better suited for treatment planning tasks that demand precise guideline adherence, such as those required in head and neck oncology.

Although the overall staging accuracy between the models was comparable (*p* = 0.83), the variation in case-specific misclassifications suggests that each model may rely on different reasoning patterns when interpreting complex clinical data. The presence of distinct non-overlapping errors highlights the potential benefit of integrating complementary AI systems or employing human oversight to mitigate individual model limitations.

The most frequently seen head and neck cancers are oral cavity and larynx cancers. Sinonasal tumours, mucosal melanomas, and primary head and neck cancers of unknown origin are seen less often [1, 17–19]. Accordingly, fewer questions related to these uncommon head and neck cancer subsites were included in the study.

Both models performed well in common cancers such as laryngeal and oral cavity malignancies, which comprised the largest proportion of the cases. In laryngeal cancer scenarios, ChatGPT achieved 66.7% accuracy in staging and DeepSeek 53.3%. Although DeepSeek slightly outperformed ChatGPT in treatment planning, this result was not statistically significant (73.3% vs. 66.7%, *p* = 0.69). Similarly, for oral cavity cancers, DeepSeek provided a higher proportion of fully correct treatment recommendations, but this result was not statistically significant (66.7% vs. 50%, *p* = 0.40). A previous study evaluating ChatGPT with 24 clinical otolaryngology questions reported that the model achieved the highest accuracy in head and neck oncology, whereas its lowest accuracy was observed in pediatric otolaryngology tasks [20]. This result is supported by other publications in the literature, which have demonstrated discrepancies in ChatGPT performance across various medical fields [20–23], as well as considerable variability in accuracy rates [24, 25], including specific documentation within otolaryngology [26–29]. Although methodological differences exist among these prior studies, the variability of ChatGPT performance underscores the importance of targeted investigations aimed at rigorously assessing the reliability and clinical applicability of artificial intelligence tools within individual head and neck cancer subsites. The comparatively smaller number of clinical scenarios generated for less common cancers within the current study, such as sinonasal malignancies, mucosal melanomas, and cancers of unknown primary origin, prevented a statistical comparison. Future studies should focus on comparing the staging and treatment success of AI tools with more questions for each head and neck cancer subsite. In this way, deficiencies in the training datasets will be able to be determined, and with their development, performance in head and neck cancers will be able to be increased.

The results of this study showed that ChatGPT incorrectly staged 40% of the cases and provided partially correct treatment recommendations in 38% of scenarios, while DeepSeek also demonstrated a substantial error rate with incorrect staging in 38% of the cases and partially correct treatment plans in 20% of scenarios. Such high error rates are particularly concerning in the context of head and neck oncology, where clinical decisions significantly impact patient survival and quality of life. These findings highlight the need for further improvement and refinement of artificial intelligence tools intended for clinical decision support, in alignment with recommendations from previous literature [29, 30].

This study provides valuable insights into the comparative performance of two advanced LLMs, ChatGPT o1 and DeepSeek V3, specifically within head and neck cancer staging and treatment planning. Although staging accuracy was comparable between the models, DeepSeek V3 exhibited superior performance in treatment recommendations, suggesting that it has potential as a practical and accessible clinical decision-support tool. Nevertheless, it is essential to emphasize that these AI systems, even when optimized through targeted dataset training and rigorous clinical validation, should serve strictly as adjunctive aids under human supervision rather than replacements for MDTs. Moreover, considering ongoing concerns regarding patient confidentiality and data security with publicly accessible AI platforms, future research and clinical integration should prioritize the development of locally hosted and secure institution-specific AI systems as recommended by Buhr et al. [14].

This study had several limitations that should be acknowledged. First, the clinical scenarios utilized in this investigation were hypothetical and limited in number; therefore, they were not fully representative of the diverse spectrum encountered in routine clinical practice. Furthermore, the distribution of cases across different head and neck cancer subsites was not uniform, potentially influencing the statistical robustness and generalizability of the current study findings. A further limitation was that the evaluation of AI responses was based solely on textual input and did not incorporate multimodal data such as imaging or pathology. That the clinical scenarios were presented to each artificial intelligence model only once, and so potential variability or inconsistency in responses upon repeated questioning could not be assessed, constituted a further limitation. Finally, although the accuracy of AI-generated responses was independently evaluated by two otolaryngologists based on NCCN Guidelines, inter-rater reliability between these evaluators was not formally assessed, introducing a potential source of subjective bias into the results.

## Conclusion

Both ChatGPT o1 and DeepSeek V3 demonstrated promising capabilities for clinical decision support in head and neck oncology, with DeepSeek V3 showing superior performance in treatment recommendations. While these artificial intelligence tools cannot replace MDTs, they can significantly streamline clinical workflows by rapidly organizing patient data, thereby enhancing board efficiency. Given the observed variations in model inaccuracies and ongoing concerns regarding patient data privacy, future efforts should prioritize the development and integration of secure, institution-specific, local LLMs tailored for oncological decision-making.

## Supplementary Information

Below is the link to the electronic supplementary material.

Supplementary file1 (DOCX 33 KB)

Supplementary file2 (DOCX 27 KB)

## Data Availability

Data are available upon reasonable request from the corresponding author.

## References

[1] Schmidl B, Hütten T, Pigorsch S, Stögbauer F, Hoch CC, Hussain T, Wollenberg B, Wirth M (2024) Assessing the use of the novel tool Claude 3 in comparison to ChatGPT 4.0 as an artificial intelligence tool in the diagnosis and therapy of primary head and neck cancer cases. Eur Arch Otorhinolaryngol 281(11):6099–6109. 10.1007/s00405-024-08828-1 39112556 10.1007/s00405-024-08828-1 PMC11512878

[2] Amin MB, Edge SB, American Joint Committee on Cancer (2017) AJCC Cancer Staging Manual. 8th ed. New York, NY: Springer

[3] National Comprehensive Cancer Network® (NCCN®) Guidelines Version 2.2025. https://www.nccn.org/professionals/physician_gls/pdf/head-and-neck.pdf. Accessed Feb 2025

[4] Cascella M, Montomoli J, Bellini V, Bignami E (2023) Evaluating the feasibility of ChatGPT in healthcare: An analysis of multiple clinical and research scenarios. J Med Syst 47(1):33. 10.1007/s10916-023-01925-4 36869927 10.1007/s10916-023-01925-4 PMC9985086

[5] Sufi F (2024) Generative pre-trained transformer (GPT) in research: A systematic review on data augmentation. Information 15(2):99. 10.3390/info15020099

[6] Berardi R, Morgese F, Rinaldi S, Torniai M, Mentrasti G, Scortichini L, Giampieri R (2020) Benefits and limitations of a multidisciplinary approach in cancer patient management. Cancer Manag Res 12:9363–9374. 10.2147/CMAR.S220976 33061625 10.2147/CMAR.S220976 PMC7533227

[7] Luchini C, Lawlor RT, Milella M, Scarpa A (2020) Molecular tumour boards in clinical practice. Trends Cancer 6(9):738–744. 10.1016/j.trecan.2020.05.008 32517959 10.1016/j.trecan.2020.05.008

[8] Thenappan A, Halaweish I, Mody RJ, Smith EA, Geiger JD, Ehrlich PF, Jasty Rao R, Hutchinson R, Yanik G, Rabah RM, Heider A, Stoll T, Newman EA (2017) Review at a multidisciplinary tumour board impacts critical management decisions of pediatric patients with cancer. Pediatr Blood Cancer 64(2):254–258. 10.1002/pbc.26201 27578484 10.1002/pbc.26201

[9] Smiju IS, Adinath DR (2025) Advancements in AI-powered NLP models: a critical analysis of ChatGPT and DeepSeek. SSRN. 10.2139/ssrn.5125445

[10] Lechien JR, Chiesa-Estomba CM, Baudouin R, Hans S (2024) Accuracy of ChatGPT in head and neck oncological board decisions: preliminary findings. Eur Arch Otorhinolaryngol 281(4):2105–2114. 10.1007/s00405-023-08326-w 37991498 10.1007/s00405-023-08326-w

[11] Lechien JR, Rameau A (2024) Applications of ChatGPT in otolaryngology-head neck surgery: A state of the art review. Otolaryngol Head Neck Surg 171(3):667–677. 10.1002/ohn.807 38716790 10.1002/ohn.807

[12] Schmidl B, Hütten T, Pigorsch S, Stögbauer F, Hoch CC, Hussain T, Wollenberg B, Wirth M (2024) Assessing the role of advanced artificial intelligence as a tool in multidisciplinary tumor board decision-making for primary head and neck cancer cases. Front Oncol 14:1353031. 10.3389/fonc.2024.1353031 38854718 10.3389/fonc.2024.1353031 PMC11157509

[13] Lukac S, Dayan D, Fink V, Leinert E, Hartkopf A, Veselinovic K, Janni W, Rack B, Pfister K, Heitmeir B, Ebner F (2023) Evaluating ChatGPT as an adjunct for the multidisciplinary tumor board decision-making in primary breast cancer cases. Arch Gynecol Obstet 308(6):1831–1844. 10.1007/s00404-023-07130-5 37458761 10.1007/s00404-023-07130-5 PMC10579162

[14] Buhr CR, Ernst BP, Blaikie A, Smith H, Kelsey T, Matthias C, Fleischmann M, Jungmann F, Alt J, Brandts C, Kämmerer PW, Foersch S, Kuhn S, Eckrich J (2025) Assessment of decision-making with locally run and web-based large language models versus human board recommendations in otorhinolaryngology, head and neck surgery. Eur Arch Otorhinolaryngol 282(3):1593–1607. 10.1007/s00405-024-09153-3 39792200 10.1007/s00405-024-09153-3 PMC11890241

[15] Peng Y, Malin BA, Rousseau JF, Wang Y, Xu Z, Xu X, Weng C, Bian J (2025) From GPT to DeepSeek: Significant gaps remain in realizing AI in healthcare. J Biomed Inform 163:104791. 10.1016/j.jbi.2025.104791 39938624 10.1016/j.jbi.2025.104791 PMC12188495

[16] Mondillo G, Colosimo S, Perrotta A, Frattolillo V, Masino M (2025) Comparative evaluation of advanced AI reasoning models in pediatric clinical decision support: ChatGPT O1 vs. DeepSeek-R1. medRxiv 10.1101/2025.01.27.25321169. Accessed 28 January 2025

[17] Ascierto PA, Accorona R, Botti G, Farina D, Fossati P, Gatta G, Gogas H, Lombardi D, Maroldi R, Nicolai P, Ravanelli M, Vanella V (2017) Mucosal melanoma of the head and neck. Crit Rev Oncol Hematol 112:136–152. 10.1016/j.critrevonc.2017.01.019 28325255 10.1016/j.critrevonc.2017.01.019

[18] Yaniv D, Su SY (2023) Updates in management strategies of locally advanced sinonasal malignancy. Curr Opin Otolaryngol Head Neck Surg 31(1):39–44. 10.1097/MOO.0000000000000866 36856185 10.1097/MOO.0000000000000866

[19] Strojan P, Ferlito A, Langendijk JA, Corry J, Woolgar JA, Rinaldo A, Silver CE, Paleri V, Fagan JJ, Pellitteri PK, Haigentz M Jr, Suárez C, Robbins KT, Rodrigo JP, Olsen KD, Hinni ML, Werner JA, Mondin V, Kowalski LP, Devaney KO, de Bree R, Takes RP, Wolf GT, Shaha AR, Genden EM, Barnes L (2013) Contemporary management of lymph node metastases from an unknown primary to the neck: II. a review of therapeutic options. Head Neck 35(2):286–93. 10.1002/hed.21899 22034062 10.1002/hed.21899

[20] Tessler I, Wolfovitz A, Alon EE, Gecel NA, Livneh N, Zimlichman E, Klang E (2024) ChatGPT’s adherence to otolaryngology clinical practice guidelines. Eur Arch Otorhinolaryngol 281(7):3829–3834. 10.1007/s00405-024-08634-9 38647684 10.1007/s00405-024-08634-9

[21] Wang C, Liu S, Yang H, Guo J, Wu Y, Liu J (2023) Ethical considerations of using ChatGPT in health care. J Med Internet Res 25:e48009. 10.2196/48009 37566454 10.2196/48009 PMC10457697

[22] Alfertshofer M, Hoch CC, Funk PF, Hollmann K, Wollenberg B, Knoedler S, Knoedler L (2024) Sailing the seven seas: A multinational comparison of ChatGPT’s performance on medical licensing examinations. Ann Biomed Eng 52(6):1542–1545. 10.1007/s10439-023-03338-3 37553555 10.1007/s10439-023-03338-3 PMC11082010

[23] Vaira LA, Lechien JR, Abbate V, Allevi F, Audino G, Beltramini GA, Bergonzani M, Bolzoni A, Committeri U, Crimi S, Gabriele G, Lonardi F, Maglitto F, Petrocelli M, Pucci R, Saponaro G, Tel A, Vellone V, Chiesa-Estomba CM, Boscolo-Rizzo P, Salzano G, De Riu G (2024) Accuracy of ChatGPT-generated information on head and neck and Oromaxillofacial surgery: A multicenter collaborative analysis. Otolaryngol Head Neck Surg 170(6):1492–1503. 10.1002/ohn.489 37595113 10.1002/ohn.489

[24] Taira K, Itaya T, Hanada A (2023) Performance of the Large language model ChatGPT on the national nurse examinations in Japan: Evaluation study. JMIR Nurs 6:e47305. 10.2196/47305 37368470 10.2196/47305 PMC10337249

[25] Thirunavukarasu AJ, Hassan R, Mahmood S, Sanghera R, Barzangi K, El Mukashfi M, Shah S (2023) Trialling a large language model (ChatGPT) in general practice with the applied knowledge test: Observational study demonstrating opportunities and limitations in primary care. JMIR Med Educ 9:e46599. 10.2196/46599 37083633 10.2196/46599 PMC10163403

[26] Johnson SB, King AJ, Warner EL, Aneja S, Kann BH, Bylund CL (2023) Using ChatGPT to evaluate cancer myths and misconceptions: artificial intelligence and cancer information. JNCI Cancer Spectr 7(2):pkad015. 10.1093/jncics/pkad015 36929393 10.1093/jncics/pkad015 PMC10020140

[27] Zalzal HG, Cheng J, Shah RK (2023) Evaluating the current ability of ChatGPT to assist in professional otolaryngology education. OTO Open 7(4):e94. 10.1002/oto2.94 38020045 10.1002/oto2.94 PMC10663981

[28] Hoch CC, Wollenberg B, Lüers JC, Knoedler S, Knoedler L, Frank K, Cotofana S, Alfertshofer M (2023) ChatGPT’s quiz skills in different otolaryngology subspecialties: an analysis of 2576 single-choice and multiple-choice board certification preparation questions. Eur Arch Otorhinolaryngol 280(9):4271–4278. 10.1007/s00405-023-08051-4 37285018 10.1007/s00405-023-08051-4 PMC10382366

[29] Teixeira-Marques F, Medeiros N, Nazaré F, Alves S, Lima N, Ribeiro L, Gama R, Oliveira P (2024) Exploring the role of ChatGPT in clinical decision-making in otorhinolaryngology: a ChatGPT designed study. Eur Arch Otorhinolaryngol 281(4):2023–2030. 10.1007/s00405-024-08498-z 38345613 10.1007/s00405-024-08498-z

[30] Buhr CR, Smith H, Huppertz T, Bahr-Hamm K, Matthias C, Blaikie A, Kelsey T, Kuhn S, Eckrich J (2023) ChatGPT versus consultants: Blinded evaluation on answering otorhinolaryngology case-based questions. JMIR Med Educ 9:e49183. 10.2196/49183 38051578 10.2196/49183 PMC10731554

