# Supplementary material for: ChatGPT versus DeepSeek in head and neck cancer staging and treatment planning: guideline-based study
Source: Eur Arch Otorhinolaryngol. 2025 Jun 17;282(9):4815–24. doi: 10.1007/s00405-025-09524-4 (PMC12423241; doi:10.1007/s00405-025-09524-4)
Supplement: Supplementary file 1 — Supplementary file1 (DOCX 33 KB) [file 405_2025_9524_MOESM1_ESM.docx]

Examples of overlapping mistakes in staging

9. A 67-year-old male patient presented with a one-month history of neck swelling and subsequently underwent a deep punch biopsy of the right tonsil, which revealed basaloid squamous cell carcinoma, p16-positive. Clinical examination identified a palpable 3 cm mass at right cervical level 2, with bilateral vocal cords mobile and no other remarkable ENT findings. Contrast-enhanced CT imaging demonstrated a 50×24mm vegetative lesion originating from the right tonsillar lobe, extending laterally from the posterior pharyngeal wall, anteriorly from the tongue base, and reaching the vallecula without mucosal involvement. Additionally, a 14 mm irregularly contoured nodule was detected in the superior segment of the left lower lung lobe, with metastasis being indeterminate. PET-CT imaging revealed increased metabolic activity (SUVmax: 15.74) in a 40 × 24 mm soft tissue lesion within the right palatine tonsil, narrowing the airway from the right. A right cervical lymph node exhibited FDG uptake (SUVmax: 8), and fine-needle aspiration biopsy (FNAB) confirmed squamous cell carcinoma metastasis. Furthermore, the lung nodule in the left lower lobe exhibited increased metabolic activity (SUVmax: 6), suggesting possible metastatic involvement.

QUESTİON/ANSWER: What is the stage of tumor?

CORRECT ANSWER: T3N1M1 HPV (+) Oropharynx Carcinoma

ChatGPT o1 Answer: T2N1M1

DeepSeek V3 Answer: T2N1M1

10. A 31-year-old male patient presented with nasal breathing difficulty and left-sided neck swelling. Clinical examination revealed a vegetative mass completely covering the choana, while bilateral middle meatus appeared normal. A palpable 2x1 cm lymph node was detected at left cervical level 2, and other ENT examinations were unremarkable. PET-CT imaging revealed intense metabolic activity (SUVmax: 16.45) at the nasopharyngeal level. Additionally, 24 mm lymph node at left cervical level 2 and 15mm lymph node at right cervical level 2 exhibited increased metabolic activity (SUVmax: 6.42, 5.45). A nasopharyngeal punch biopsy and fine-needle aspiration biopsy (FNAB) from the left cervical level 2-3 mass were performed, with FNAB pathology confirming malignancy. Histopathological analysis of the nasopharyngeal biopsy confirmed nonkeratinized squamous cell carcinoma, EBV-positive.

QUESTİON/ANSWER: What is the stage of tumor?

CORRECT ANSWER: T2N2M0 Nasopharynx carcinoma

ChatGPT o1 Answer: T2N1M0

DeepSeek V3 Answer: T1N1M0

11. A 60-year-old male patient presented with a two-year history of chronic ulceration on the lower lip and subsequently underwent an incisional biopsy, which confirmed squamous cell carcinoma. Clinical examination identified a 3 × 2 cm ulcerated lesion in the midline of the lower lip, extending to the inner mucosa without submucosal invasion beyond the vermillion line. No palpable lymphadenopathy (LAP) was detected in the neck, and other ENT examinations were unremarkable. Contrast-enhanced CT imaging revealed asymmetric mucosal thickening in the midline and left lower lip without evidence of destruction of the adjacent bone structure. PET-CT imaging demonstrated increased metabolic activity (SUVmax: 8.40) localized solely to the primary tumor site, with no evidence of pathological FDG uptake elsewhere in the body.

QUESTİON/ANSWER: What is the stage of tumor?

CORRECT ANSWER: T2N0M0 Oral Cavity (Lip) Squamous cell carcinoma

ChatGPT o1 Answer: T1N0M0

DeepSeek V3 Answer: T1N0M0

14.A 58-year-old male patient presented with a 2.5-month history of progressive hoarseness and dysphagia that did not resolve. İndirect laryngoscopic evaluation revealed a thickened, edematous band extending from the left arytenoid to the contralateral arytenoid, along with a smooth-surfaced mass lesion filling and bulging the ventricle from the left arytenoid, extending to the anterior commissure, and causing edema in the vocal cord. PET-CT imaging demonstrated pathologically increased F-18 FDG uptake in a 5.5 cm segment of the left supraglottic-glottic region (SUVmax: 13.49), involving the left epiglottic fold, left piriform sinus, left false and true vocal cords, and obliterating the left lateral ventricle. Additionally, the lesion’s borders with the esophagus were indistinct, and pathological FDG uptake (SUVmax: 11.23) was observed. Increased metabolic activity was also noted in a lymph node (~3,5 × 2 cm) at left cervical level 2 (SUVmax: 5.49). Histopathological analysis of the biopsy confirmed squamous cell carcinoma. Additionally, fine-needle aspiration biopsy (FNAB) of the left level 2 lymph node confirmed metastatic squamous cell carcinoma.

QUESTİON/ANSWER: What is the stage of tumor?

CORRECT ANSWER: T3N2aM0 Hypopharynx Carcinoma

ChatGPT o1 Answer: T4aN2aM0

DeepSeek V3 Answer: T4aN2bM0

17. A 68-year-old woman presented with a six-month history of a sore on the lower lip, and clinical examination revealed a crusted, ulcerovegetating mass of approximately 3x2 cm on the right half of the lower lip, with no palpable cervical lymphadenopathy and otherwise normal ENT findings. Biopsy confirmed well-differentiated microinvasive squamous cell carcinoma. A contrast-enhanced CT scan of the neck and thorax revealed contour irregularity and increased mucosal contrast enhancement in the soft tissue of the right half of the lower lip.

No pathological lymphadenopathy was observed in the neck.

QUESTİON/ANSWER: What is the stage of tumor?

CORRECT ANSWER: T2N0M0 lower lip squamous cell carcinoma

ChatGPT o1 Answer: T1N0M0

DeepSeek V3 Answer: T1N0M0

19.A 71-year-old male patient presented with a two-month history of neck swelling. Laryngoscopic examination revealed a lesion originating from the laryngeal surface of the epiglottis petiolus, extending to but not involving the anterior commissure, while bilateral vocal cords, the subglottic area, piriform sinuses, and ventricles appeared normal. There was a 1x1 cm palpable lymphadenopathy at right level 2B and a 1x1 cm palpable lymphadenopathy at left level 4, with otherwise normal ENT findings. Pathological analysis confirmed squamous cell carcinoma with basaloid morphology. PET imaging demonstrated focal pathological FDG uptake in a 1 cm nodular density at the anterior commissure of the larynx (SUVmax: 12.74), as well as pathological uptake in a 2.5x2 cm lymph node in the right superior jugular region (SUVmax: 12.45), multiple right posterior cervical lymph nodes (SUVmax: 6), and a 2x1.8 cm lymph node in the left level 2 cervical region (SUVmax: 15.63), with adjacent millimetric lymph nodes showing no distinct pathological uptake. No pathological FDG uptake was observed in other body regions on PET-CT.

QUESTİON/ANSWER: What is the stage of tumor?

CORRECT ANSWER: T1N2cM0 Supraglottic Laryngeal Carcinoma

ChatGPT o1 Answer: T2N2cM0

DeepSeek V3 Answer: T2N2cM0

27.A 60-year-old male patient with a six-month history of shortness of breath underwent direct laryngoscopy and biopsy with pathology confirming keratinized squamous cell carcinoma. Laryngoscopic examination revealed an ulcerovegetating mass destructing the right half of the epiglottis and extending to the left half, involving the vallecula, tongue root, bilateral aryepiglottic folds, band ventricles, and the epiglottis petiolus. A 2x2 cm mobile, regularly circumscribed palpable lymph node was detected at left level 2. The right vocal cord was mobile, while the left vocal cord could not be evaluated due to the mass. Other ENT examinations were normal. PET imaging demonstrated increased metabolic activity (SUVmax: 10.73) in a soft tissue lesion filling the preepiglottic space anteriorly paraglottic space on the right, with continuity extending up to the level of the false vocal cord on the right. İncreased FDG uptake was observed in cervical lymph nodes at right levels 2A and 3, the largest measuring 20x6 mm (SUVmax: 7.43), and in a 15x5 mm lymph node at left level 3 (SUVmax: 8.13). What should be the optimal treatment approach for this patient?

QUESTİON/ANSWER: What is the stage of tumor?

CORRECT ANSWER: T3N2cM0 Supraglottic Laryngeal Carcinoma

ChatGPT o1 Answer: T4aN2cM0

DeepSeek V3 Answer: T4aN2cM0

31.A 63-year-old male patient with a two-month history of sores and discharge on the left side of the neck underwent direct laryngoscopy and biopsy after a suspicious lesion was observed in the hypopharynx, with pathology confirming HPV-positive squamous cell carcinoma. Examination revealed dense purulent secretion at the level of the tongue root, vallecula, piriform sinuses, and band ventricle, leading to suboptimal evaluation, where left vocal cord movement was limited while right vocal cord movement was observed, with bilateral arytenoid edema and adequate rim patency. A 1 cm fistulized area with saliva and purulent discharge was noted at left level 3, while other ENT examinations were normal. Follow-up imaging showed regression, and a biopsy from the fistulized area on the left neck skin confirmed squamous cell carcinoma. PET-CT findings demonstrated a 59x42 mm mass lesion at the left oropharyngeal level, extending inferiorly into the larynx and hypopharynx, with intense FDG uptake (SUVmax: 27.49), involving the left piriform sinus and invading the left internal carotid artery. Additionally, lymph nodes at right level 1A (8x6 mm, SUVmax: 5.74) and left level 3 (9x5 mm, SUVmax: 2.77) exhibited low metabolic activity, while no pathological FDG uptake was detected elsewhere in the body, and all systemic scans were normal.

QUESTİON/ANSWER: What is the stage of tumor?

CORRECT ANSWER: T4N2M0 Oropharynx Carcinoma

ChatGPT o1 Answer: T4aN1M0

DeepSeek V3 Answer: T4aN1M0

32.A 50-year-old male patient with a 1.5-month history of an intraoral mass underwent a liver biopsy performed by the Gastroenterology Department due to the simultaneous onset of abdominal pain, revealing a poorly differentiated malignant epithelial tumor, while a punch biopsy from the oral cavity confirmed an undifferentiated malignant tumor. On physical examination, a 6×5 cm ulcerovegetative mass was noted in the right buccal region, causing gingival displacement and trismus, while the nasopharynx and laryngeal examination were normal, with no palpable cervical lymphadenopathy. PET-CT imaging demonstrated intense FDG uptake (SUVmax: 12.11) in a lesion in the right maxillary region, causing destruction of the zygomatic bone and right half of the maxilla, extending subcutaneously, invading the right half of the mandible, and measuring 72×43 mm at its widest dimension. Additionally, increased metabolic activity (SUVmax: 6.66) was noted in a focal nodular area at the level of the anterior aspect of the first rib, involving the pleura-muscle structure in the apical segment of the upper lobe of the right lung, with invasion of the anterior aspect of the first rib. The liver appeared enlarged, and multifocal FDG-avid lesions (SUVmax: 11) were observed, encompassing almost the entire right hepatic lobe from the diaphragmatic level, with invasion into the caudate lobe and the right perirenal area, reaching a maximum dimension of 186×185 mm, and exhibiting areas of necrosis. No additional pathological FDG uptake was detected in other body regions.

QUESTİON/ANSWER: What is the stage of tumor?

CORRECT ANSWER: T4aNoM1 Oral Cavity Carcinoma

ChatGPT o1 Answer: T4bN0M1

DeepSeek V3 Answer: T4bN0M1

41.A 41-year-old male patient presented with hoarseness and respiratory distress for four months and underwent direct laryngoscopy, biopsy, and tracheotomy following the detection of a laryngeal lesion. Postoperative pathology confirmed keratinized squamous cell carcinoma, HPV-negative. Laryngoscopic examination revealed a vegetating mass originating from the posterior tongue root, forming a cavity in the vallecula, destroying the entire epiglottis, and submucosally involving the right band ventricle and the right aryepiglottic fold, while extending to the anterior mucosa of the left arytenoid without direct involvement of the arytenoid itself. Vocal cord mobility could not be clearly assessed. On bilateral level 2, hard, fixed lymph nodes measuring approximately 4×2 cm were noted, covered with normal skin. PET-CT findings demonstrated pathological FDG uptake in multiple bilateral jugular and deep cervical lymph nodes, some measuring up to 4.5 cm in diameter, with central hypometabolic (necrotic?) areas (SUVmax: 23.73). In the larynx, a pathological increase in FDG uptake (SUVmax: 30.56) was observed in an annular tumor thickening extending superiorly to the supraglottic level and inferiorly to the right vocal cord, causing significant airway narrowing. Additionally, prevertebral fascia invasion was noted. No pathological FDG uptake was detected in other body regions on PET-CT.

QUESTİON/ANSWER: What is the stage of tumor?

CORRECT ANSWER: T4bN3bM0 Supraglottic Larynx Carcinoma

ChatGPT o1 Answer: T4bN2cM0

DeepSeek V3 Answer: T4bN2cM0

43.An 84-year-old male patient with a two-year history of a vegetative lesion in the right buccal region, initially presenting with redness, underwent an incisional biopsy, which confirmed non-keratinized squamous cell carcinoma of the right cheek mucosa. On clinical examination, a vegetative lesion originating from the upper gingiva was observed, extending inferiorly to the lower gingiva, posteriorly from the retromolar trigone, and anteriorly beyond the oral commissure, infiltrating past the commissure. A fixed palpable lymph node was detected at right cervical level 2, while other ENT examinations were unremarkable. Contrast-enhanced imaging revealed right pterygoid plate involvement. PET-CT imaging demonstrated increased metabolic activity (SUVmax: 12.58) in the right cheek lesion, which extended from the infraorbital level into the maxillary right half, filling the subcutaneous tissue. Additionally, a hypermetabolic 4 cm lymph node (SUVmax: 9.63) was observed at right cervical level 2. The cheek lesion was indistinguishable from the hard palate and raised suspicion of invasion into the dental root.

QUESTİON/ANSWER: What is the stage of tumor?

CORRECT ANSWER: T4bN3bM0 Oral Cavity Carcinoma

ChatGPT o1 Answer: T4bN2M0

DeepSeek V3 Answer: T4bN2M0

47.A 47-year-old male patient presented with a 2 year history of nasal obstruction predominantly affecting the left side. Endoscopic examination revealed a mass originating from the lower end of the left middle turbinate and extending to the upper end of the inferior turbinate, partially obstructing the nasal passage and preventing visualization of the choana, with seropurulent discharge present. The right nasal passage was normal. MRI findings demonstrated a lobulated mass causing nasal passage obliteration, with indistinct borders from the turbinates and maxillary sinus ostium, exhibiting a heterogeneous T2 hyperintense signal and cerebriform contrast enhancement after IVKM injection. The lesion appeared to cause bone erosion at this level and was suspected to be an inverted papilloma. However, puch biopsy confirmed squamous cell carcinoma (SCC).

QUESTİON/ANSWER: What is the stage of tumor?

CORRECT ANSWER: T2N0M0 Nasal Cavitiy Squamous Cell Carcinoma

ChatGPT o1 Answer: T3N0M0

DeepSeek V3 Answer: T3N0M0
