# Supplementary material for: ChatGPT versus DeepSeek in head and neck cancer staging and treatment planning: guideline-based study
Source: Eur Arch Otorhinolaryngol. 2025 Jun 17;282(9):4815–24. doi: 10.1007/s00405-025-09524-4 (PMC12423241; doi:10.1007/s00405-025-09524-4)
Supplement: Supplementary file 2 — Supplementary file2 (DOCX 27 KB) [file 405_2025_9524_MOESM2_ESM.docx]

**Examples of non-overlapping mistakes in staging**

**1.**A 63-year-old female patient with no known comorbidities presented to the emergency department with respiratory distress, and emergency evaluation revealed significant airway obstruction due to a large supraglottic mass, necessitating an urgent tracheotomy for airway stabilization. Direct laryngoscopic examination identified an ulcerovegetative mass involving the right half of the epiglottis, extending to the interarytenoid space, destroying the band ventricle, and affecting the bilateral band ventricles, anterior commissure, right vocal cord, and the medial wall of the right pyriform sinus submucosally. The right vocal cord was paralytic, while the left vocal cord remained mobile, and other ENT examinations were unremarkable. Contrast-enhanced neck CT revealed a soft tissue-dense lesion measuring approximately 45 × 20 mm at the level of the epiglottis, involving the right aryepiglottic fold and causing slight airway narrowing. PET-CT findings demonstrated intense metabolic activity (SUVmax: 24.73) in the mass lesion invading the right half of the epiglottis, the right pyriform sinus in the supraglottic region, and extending to the anterior commissure, right vocal cord, and right arytenoid cartilage at the level of the vocal cords. Histopathological examination confirmed squamous cell carcinoma.

QUESTİON/ANSWER: What is the stage of tumor?

Correct Answer: **T3N0M0** Larynx Carcinoma

ChatGpt o1 Answer: T4aN0M0

DeepSeek V3 Answer: T3N0M0

**7.**A 35-year-old male patient presented with nasal breathing difficulty and subsequently underwent a punch biopsy of the nasopharynx, which revealed nonkeratinized squamous cell carcinoma, undifferentiated subtype, EBV-positive. CT imaging demonstrated a polypoid soft tissue density in the right middle turbinate at the level of the choana, hypertrophy of the right inferior turbinate, and narrowing of the right middle meatus. Given these findings, a PET scan was performed for further evaluation. PET-CT revealed a hypermetabolic lesion in the nasopharynx, measuring 43 × 41 × 29 mm (SUVmax: 11.79), located in the right lateral and posterosuperior region, extending across the midline to the left, consistent with the primary tumor. Additionally, bilateral sphenoethmoidal recesses were obliterated with soft tissue. Heterogeneous FDG uptake was observed in the anterolateral bony structures of the right occipitotemporal region at the skull base (SUVmax: 6.65), raising suspicion for metastasis. Furthermore, a lytic lesion with diffusely increased FDG uptake (SUVmax: 11.20) was detected in the C7 vertebral body, suggestive of metastatic involvement.

QUESTİON/ANSWER: What is the stage of tumor?

Correct Answer: **T3N2M1** Nasopharynx Carcinoma

ChatGpt o1 Answer: T3N2M1

DeepSeek V3 Answer: T3N0M1

**20**.A 67-year-old male patient presented with a one-month history of neck swelling, and a deep punch biopsy of the right tonsil confirmed squamous cell carcinoma with p16 negativity. PET imaging revealed a vegetative lesion originating from the right tonsillar lodge, extending laterally from the posterior pharyngeal wall and inferiorly from the root of the tongue toward the vallecula without mucosal involvement. The epiglottis and left tonsil were normal, and bilateral vocal cords were mobile. Clinical examination identified a palpable swelling of approximately 3 cm at right level 2, with otherwise normal ENT findings. PET imaging demonstrated increased metabolic activity in a 39x37 mm soft tissue lesion (SUVmax: 15.74) in the right palatine tonsil. The 3 cm lymph node at right level 2 showed FDG uptake with SUVmax: 8, and no other pathological FDG uptake was detected in the rest of the body on PET-CT.

QUESTİON/ANSWER: What is the stage of tumor?

Correct Answer: **T3N1M0** HPV (-) Oropharyngeal Carcinoma

ChatGpt o1 Answer: T3N1M0

DeepSeek V3 Answer: T4N1M0

**22.**A 54-year-old male patient with a 12-month history of hoarseness underwent direct laryngoscopy and biopsy in our center on 02.12.2024. Laryngoscopic examination revealed an easily accessible airway and a mass lesion involving the left band ventricle, extending toward the right band ventricle, involving the vocal cord with anterior and medial subglottic extension of approximately 1.2 cm, and extending posteriorly into the subglottic area from the inferior aspect of the interarytenoid region. The epiglottis, tongue root, vallecula, and piriform sinuses were intact. The left vocal cord was fixed, while the right vocal cord was mobile. No palpable lymphadenopathy was detected in the neck, and other ENT examinations were unremarkable. Pathology confirmed keratinized squamous cell carcinoma. PET imaging demonstrated asymmetric thickening of the left vocal cord with increased metabolic activity extending to the anterior commissure and subglottic level (SUVmax: 11.29). MRI findings revealed a lobule-contoured mass lesion originating from the free surface of the epiglottis and preepiglottic adipose tissue on the left, extending to the posterior commissure, involving the false and true vocal cords at the level of the aryepiglottic fold, obliterating the left vallecula, extending to the left subglottic area, and invading the paraglottic adipose tissue. The lesion appeared hyperintense on T2-weighted imaging, exhibited heterogeneous contrast enhancement following IV contrast administration, and showed diffusion restriction. No pathological uptake was detected in other body regions on PET-CT.

QUESTİON/ANSWER: What is the stage of tumor?

Correct answer: **T3N0M0** Transglottic Laryngeal Carcinoma

ChatGpt o1 Answer: T3N0M0

DeepSeek V3 Answer: T4aN0M0

**23.** A 73-year-old male patient with a history of coronary artery bypass surgery five months ago presented with a one-year history of hoarseness and subsequently underwent direct laryngoscopy and biopsy, with postoperative pathology confirming squamous cell carcinoma involving the epiglottis and anterior commissure. Laryngoscopic examination revealed a difficult airway, and 0-degree endoscopic mucosal evaluation demonstrated a 0.3x0.3 cm mucosal lesion in the anterior left band ventricle, while the bilateral vocal cords, arytenoids, epiglottis, right band ventricle, tongue root, vallecula, piriform sinuses, and subglottic area appeared intact with preserved vocal cord mobility. No palpable lymphadenopathy was detected in the neck, and other ENT findings were unremarkable. PET imaging demonstrated focal FDG uptake (SUVmax: 20.67) in a 14x10x15 mm lobulated soft tissue-dense lesion extending from the left inferior epiglottis to the left true vocal cord at the supraglottic level, with a polypoid extension toward the superior lumen and thickening of the left aryepiglottic fold. MRI revealed tumor invasion of the preepiglottic space and involvement of the inner lamina of the thyroid cartilage. Additionally, PET-CT identified a 3 cm lymph node with FDG uptake (SUVmax: 8) in the left cervical region, and fine-needle aspiration biopsy confirmed squamous cell carcinoma. No pathological FDG uptake was detected in other body regions on PET-CT.

QUESTİON/ANSWER: What is the stage of tumor?

Correct answer: **T3N1M0** Supraglottic Laryngeal Carcinoma

ChatGpt o1 Answer: T3N1M0

DeepSeek V3 Answer: T4aN1M0

**28.**A 65-year-old male patient with hoarseness for 3 months underwent direct laryngoscopy and biopsy, revealing squamous cell carcinoma, papillary subtype. Laryngoscopic examination showed a left-dominant 2x1 cm ulcerovegetative mass lesion in the midline of the epiglottic endolaryngeal surface, with intact bilateral ventricular folds, petiole, ventricles, anterior commissure, vocal cords, piriform sinuses, tongue base, vallecula, and subglottic area; vocal cords were bilaterally mobile, and no palpable cervical lymphadenopathy was detected. PET imaging demonstrated a 26x22 mm supraglottic lesion extending to the paraepiglottic area on the right of the midline with increased metabolic activity (SUVmax: 6.1), and a subcentimetric lymph node at level 2A on the right with low-level metabolic activity (SUVmax: 1.77). CT scan revealed an irregularly contoured 30x20x20 mm supraglottic solid mass extending to the epiglottis, glossoepiglottic fold, bilateral aryepiglottic folds, and right paraglottic space, causing airway narrowing; no cortical erosion or focal sclerosis was observed in the thyroid cartilage lamina. Pulmonary function tests indicated good lung capacity.

QUESTİON/ANSWER: What is the stage of tumor?

Correct answer: **T3N0M0** Supraglottic Laryngeal Carcinoma

ChatGpt o1 Answer: T2N0M0

DeepSeek V3 Answer: T3N0M0

**29.** A 60-year-old male patient presented with a six-month history of swelling in the left neck and a wound in the mouth. Clinical examination revealed fullness in the nasopharynx and posterior oropharyngeal wall with smooth mucosal coverage. A leukoplastic ulcerovegetating mass lesion was observed, originating from the right retromolar trigone and extending to the right tonsil, soft palate, uvula, and right tongue root. Radiological imaging detected a 2x2 cm hard lymph node at right level 2 and a 1x1 cm hard lymph node at left level 2. In the nasopharynx, a mass lesion involving the torus tubarius and uvula was noted, measuring 18 mm at its thickest part, with irregularly contoured palatine tonsils almost completely obliterating the oropharyngeal air column at the level of the tonsils. The lesion extended into the parapharyngeal fat planes on the right and invaded the right lateral tongue root. PET-CT findings included a central cystic necrotic lymph node (18 mm) at right level 2A and multiple lobulated lymph nodes at left level 2B, the largest measuring 20x14 mm. The primary tumor exhibited intense FDG uptake (SUVmax: 20.5) in a 41x26 mm mass extending from the right lateral nasopharynx to both lateral oropharyngeal regions and the right palatine tonsil. Intense FDG uptake (SUVmax: 11.1) was observed in the right retropharyngeal, left parotid lobe, left cervical level 2B, and right cervical level 2A lymph nodes, the largest measuring 21x16 mm. No other pathological FDG uptake was detected in other body regions. Initial tru-cut biopsy of the left cervical level 2B lymph node was reported as reactive lymph node, prompting further evaluation. Subsequent nasopharyngeal punch biopsy and incisional biopsy from the oropharyngeal lesion confirmed keratinized squamous cell carcinoma, HPV-negative, in the oropharynx, while the nasopharyngeal biopsy showed reactive lymphoepithelial tissue.

QUESTİON/ANSWER: What is the stage of tumor?

Correct answer**: T4N2cM0** HPV (-) Oropharynx Carcinoma

ChatGpt o1 Answer: T4N2bM0

DeepSeek V3 Answer: T4N2cM0

**30.** A 68-year-old male patient with a two-year history of right ear pain and a four- to five-month history of right neck swelling underwent a tru-cut biopsy of the right level 2 lymph node, confirming metastasis of keratinized squamous cell carcinoma, with EBV and HPV reported as negative. PET-CT findings demonstrated a 31x26 mm lymph node at right cervical level 2A to level 3 with FDG uptake (SUVmax: 7.9) and bilateral cervical lymph nodes at level 2B and level 3, with a maximum dimension of 2 cm, showing FDG uptake (SUVmax: 5.3). No pathological FDG uptake was detected in other body regions on PET-CT, and all systemic scans were normal. Direct laryngoscopy and nasopharyngoscopy were normal, and a nasopharyngeal biopsy, bilateral tonsillectomy, and tongue base resection were performed, with pathology results reported as normal. Additionally, a comprehensive head and neck skin and mucosal examination was normal.

QUESTİON/ANSWER: What is the stage of tumor?

Correct answer**: T0N2cM0** Carcinoma of Unknown Primary

ChatGpt o1 Answer: T0N2bM0

DeepSeek V3 Answer: T0N2cM0

**37.** A 49-year-old male patient was referred to an external center with a complaint of swelling in the right neck, and nasopharyngeal punch biopsy confirmed non-keratinized squamous cell carcinoma, differentiated subtype, EBV positive, in the nasopharynx. On physical examination, a purulent vegetating mass originating from the right Rosenmüller fossa was observed, covering 50% of the choana, with the right tympanic membrane intact but dull, and a 3×4 cm lymph node palpable at right level 2. CT imaging revealed an asymmetric soft tissue mass measuring approximately 32×23 mm in the right posterior lateral wall of the nasopharynx, causing narrowing of the nasopharyngeal airway column. Additionally, mild cortical erosion was noted in the right anterior clivus, raising suspicion for bone invasion. PET-CT demonstrated increased FDG uptake in the right servical lymph node (SUVmax: 11.2) and in the primary tumor site (SUVmax: 20).

QUESTİON/ANSWER: What is the stage of tumor?

Correct answer**: T3N1M0** Nasopharyngeal Carcinoma

ChatGpt o1 Answer: T3N2M0

DeepSeek V3 Answer: T3N1M0

**38.**A 55-year-old male patient with a history of tobacco and alcohol use presented with a non-healing ulcerative lesion (2×1 cm) on the right half of the tongue persisting for four weeks. On bimanual palpation, a firm, tender lesion was identified, with no palpable cervical lymphadenopathy. MRI revealed a heterogeneously contrast-enhancing 2 cm lesion without suspicious cervical lymphadenopathy. Incisional biopsy confirmed squamous cell carcinoma (SCC), and the patient underwent tumor excision of the tongue and left level 1-5 selective neck dissection. Postoperative pathology demonstrated a 1.9×1 cm SCC focus, a depth of invasion (DOI) of 12 mm, and a closest surgical margin of 6 mm. Perineural invasion (PNI) and lymphovascular invasion (LVI) were reported as positive. Neck dissection revealed a single 0.8 cm intranodal SCC metastasis in right level 2 without extracapsular extension (ECE).

QUESTİON/ANSWER: What is the stage of tumor?

Correct answer**:** **pT3N1M0** Oral Cavity (tongue) Carcinoma

ChatGpt o1 Answer: T2N1M0

DeepSeek V3 Answer: T3N1M0

**39.** A 55-year-old male patient presented with a 3- to 4-month history of bilateral neck swelling. Endoscopic examination was performed, and a nasopharyngeal punch biopsy revealed nasopharyngeal carcinoma, consistent with non-keratinized squamous cell carcinoma. Physical examination identified a hard, fixed, conglomerated 5×5 cm lymph node mass filling level 5 on the right side of the neck and a 7×7 cm lymph node mass filling level 5 on the left side of the neck. A crusted lesion was observed in the midline of the nasopharynx, while other ENT examinations were unremarkable. MRI findings revealed that the left-sided mass extended into the left parapharyngeal space. PET-CT findings demonstrated posterior nasopharyngeal wall thickening with increased FDG uptake (SUVmax: 17.08), reaching approximately 2 cm at its thickest point, more prominent on the right side. Additionally, a centrally necrotic lymph node measuring 42×32 mm in the left upper jugular area exhibited intense metabolic activity (SUVmax: 15.54). Further metabolic activity uptake was observed in the left middle-lower jugular (SUVmax: 4.28) and posterior cervical lymph nodes (SUVmax: 9.34), the largest measuring approximately 8 mm. On the right side, a lymph node in the upper jugular region measuring 27×17 mm showed intense FDG uptake (SUVmax: 19.84), while a right posterior cervical lymph node approximately 9 mm in size demonstrated FDG uptake (SUVmax: 14.25).

QUESTİON/ANSWER: What is the stage of tumor?

Correct answer**:** **T2N3bM0** Nasopharyngeal Carcinoma

ChatGpt o1 Answer: T2N3bM0

DeepSeek V3 Answer: T3N3bM0

**40.** A 68-year-old male patient presented with hoarseness and underwent direct laryngoscopy (D/L) with biopsy, which confirmed squamous cell carcinoma (SCC). He subsequently received 29 fractions of radiotherapy (RT). During follow-up, the patient developed respiratory distress, and D/L with biopsy was repeated, revealing a lesion suggestive of recurrence. The patient then underwent total laryngectomy, bilateral level 2-3-4 neck dissection, and reconstruction with a pectoralis major myofascial flap following a postoperative pathology report confirming moderately differentiated SCC (pT3N2b) with perineural and perivascular invasion. He was referred to Radiation Oncology for adjuvant RT and received 4 cycles of cisplatin-based chemotherapy and 23 fractions of concurrent radiotherapy to the surgical bed and cervical lymphatics, totaling 4600 cGy.During follow-up, the patient presented with bleeding from the tracheostomy site, and an incisional biopsy from the stoma confirmed SCC. PET-CT imaging demonstrated pathological FDG uptake in the supraglottic region of the larynx at the oropharyngeal level in an annular pattern (SUVmax: 21.44), suggestive of recurrence. Additionally, the larynx appeared adhered to the skin at the mediastinal entry level (tracheostomy site?), with intense FDG uptake in this area (SUVmax: 16.63), suspicious for recurrence or an inflammatory process. Furthermore, pathological FDG uptake was observed in the right upper paratracheal and precarinal mediastinal lymph nodes, measuring approximately 1.8×1.5 cm (SUVmax: 4.19).

QUESTİON/ANSWER: What is the stage of tumor?

Correct answer**:** T4bN2M0 Unresectable, Inoperable Larynx Carcinoma

ChatGpt o1 Answer: T4bN2M0

DeepSeek V3 Answer: T4aN0M1

**42.** An 82-year-old male patient presented with a two-month history of swelling in the right infraauricular region. Fine-needle aspiration biopsy (FNAB) from the deep lobe of the right parotid gland revealed findings consistent with salivary duct carcinoma. On physical examination, a 2×2 cm hard, fixed lesion was identified in the right infraauricular region,

covered with normal skin, with no facial asymmetry or palpable cervical lymphadenopathy. Other ENT examinations were unremarkable. Radiologic evaluation (RDUS) demonstrated a 19×22 mm heterogeneous solid lesion with calcific areas and vascularization in the deep lobe of the right parotid gland. The patient underwent right total parotidectomy, right level 1b-2-3 neck dissection, and facial notch neck dissection. Postoperative pathology confirmed salivary duct carcinoma ex pleomorphic adenoma, with positive CERB-B2 and androgen receptor (AR) expression. Additionally, four metastatic lymph nodes were identified in the right total parotidectomy specimen.

QUESTİON/ANSWER: What is the stage of tumor?

Correct answer**: T2N2bM0** Salivary Gland (Parotid) Carcinoma

ChatGpt o1 Answer: T3N2bM0

DeepSeek V3 Answer: T2N2bM0

**46.** A 50-year-old male patient presented with nasal obstruction and epistaxis persisting for six months. An incisional biopsy from the left nasal passage confirmed malignant melanoma. On physical examination, a mass filling the anterior left nasal passage, originating from the medial aspect of the inferior turbinate, was observed, consistent with malignant melanoma. The patient had a congenital inward gaze restriction in the right eye, while other ENT examinations were unremarkable. PET-CT findings demonstrated metabolic activity (SUVmax: 5.64) in a soft tissue lesion measuring approximately 26×16 mm, located anteriorly in the left nasal cavity, causing airway obstruction with borders indistinguishable from the conchal mucosa.

QUESTİON/ANSWER: What is the stage of tumor?

Correct answer**: T3N0M0** Mucosal Malign Melonoma

ChatGpt o1 Answer: T1N0M0

DeepSeek V3 Answer: T3N0M0

**50.** A 60-year-old male patient underwent direct laryngoscopy (D/L) and biopsy five years ago due to hoarseness, with postoperative pathology confirming squamous cell carcinoma (SCC) of the left vocal cord and SCC in situ of the right vocal cord. He subsequently received 35 fractions of radiotherapy (RT). Upon recurrence, D/L and biopsy again confirmed SCC, leading to a total laryngectomy and left lateral neck dissection, followed by concurrent chemoradiotherapy (CRT) with 7 cycles of cisplatin and RT. During follow-up, granulation tissue around the stoma was observed, and biopsy of the lesion confirmed recurrent SCC. Postoperative PET-CT findings showed fibrotic changes in the submental, submandibular, and anterior cervical regions of the neck, with diffuse edema and mildly increased metabolic activity (SUVmax: 3.24). At the hypopharyngeal level, mucosal thickening in the cervical esophagus was noted, and post-laryngectomy changes with a tracheostomy cannula were visible. Additionally, a paratracheal soft tissue structure measuring approximately 20×11 mm inferior to the tracheostomy line exhibited increased FDG uptake (SUVmax: 7.25). Biopsy from this region confirmed keratinized squamous cell carcinoma.

QUESTİON/ANSWER: What is the stage of tumor?

Correct answer**: T4bN0M0** Larynx Carcinoma

ChatGpt o1 Answer: T4bN0M0

DeepSeek V3 Answer: T3N1M0
